# Loss of microbiota-derived protective metabolites after neutropenic fever

**DOI:** 10.1038/s41598-022-10282-0

**Published:** 2022-04-15

**Authors:** Armin Rashidi, Maryam Ebadi, Tauseef Ur Rehman, Heba Elhusseini, Hossam Halaweish, Shernan G. Holtan, Sivapriya Ramamoorthy, Daniel J. Weisdorf, Alexander Khoruts, Christopher Staley

**Affiliations:** 1 Division of Hematology, Oncology, and Transplantation, Department of Medicine, University of Minnesota, 14-100 PWB, MMC 480, 420 Delaware St. SE, Minneapolis, MN 55455 USA; 2 Department of Surgery, University of Minnesota, Minneapolis, MN USA; 3 Metabolon, Inc, Morrisville, NC USA; 4 Division of Gastroenterology, Hepatology, and Nutrition, Department of Medicine, University of Minnesota, Minneapolis, MN USA

**Keywords:** Cancer, Microbiome

## Abstract

Neutropenic fever (NF) is a common complication of chemotherapy in patients with cancer which often prolongs hospitalization and worsens the quality of life. Although an empiric antimicrobial approach is used to prevent and treat NF, a clear etiology cannot be found in most cases. Emerging data suggest an altered microbiota-host crosstalk leading to NF. We profiled the serum metabolome and gut microbiome in longitudinal samples before and after NF in patients with acute myeloid leukemia, a prototype setting with a high incidence of NF. We identified a circulating metabolomic shift after NF, with a minimal signature containing 18 metabolites, 13 of which were associated with the gut microbiota. Among these metabolites were markers of intestinal epithelial health and bacterial metabolites of dietary tryptophan with known anti-inflammatory and gut-protective effects. The level of these metabolites decreased after NF, in parallel with biologically consistent changes in the abundance of mucolytic and butyrogenic bacteria with known effects on the intestinal epithelium. Together, our findings indicate a metabolomic shift with NF which is primarily characterized by a loss of microbiota-derived protective metabolites rather than an increase in detrimental metabolites. This analysis suggests that the current antimicrobial approach to NF may need a revision to protect the commensal microbiota.

## Introduction

Many patients with cancer develop a fever during periods of chemotherapy-induced neutropenia^1^. In patients with acute myeloid leukemia (AML), antibiotic prophylaxis against neutropenic fever (NF) is only modestly successful as most patients develop NF despite prophylaxis. In addition, current treatment for NF is based on broad-spectrum antibiotics which can disrupt gut microbial communities. Features of microbiota damage in these patients include microbiota community domination^2,3^, diversity loss^4,5^, and pathogen outgrowth^6,7^. Dysbiosis in these patients has been associated with adverse clinical outcomes including *Clostridioides difficile* diarrhea^8^, neutropenic fever^9^, and infections^3,10–18^.

As the etiology of NF cannot be identified in most cases, the current antibiotic-centered approach to NF prophylaxis and treatment is largely empiric. Therefore, the complications arising from antibiotic-induced dysbiosis are partly a result of our limited understanding of NF pathogenesis^19^. In our recent work, we proposed that an altered crosstalk between the gut microbiota and host may trigger NF^9^. Our hypothesis in the present study was that the serum metabolome is altered after NF and at least some of the altered metabolites are associated with the gut microbiota. To test this hypothesis, we conducted a multi-omics analysis of the serum metabolome and gut microbiome before and after NF in patients with AML. We identified a major metabolomic shift which was unexpectedly characterized by a loss of microbiota-derived protective metabolites rather than an increase in detrimental metabolites.

## Methods

**Figure 1:**
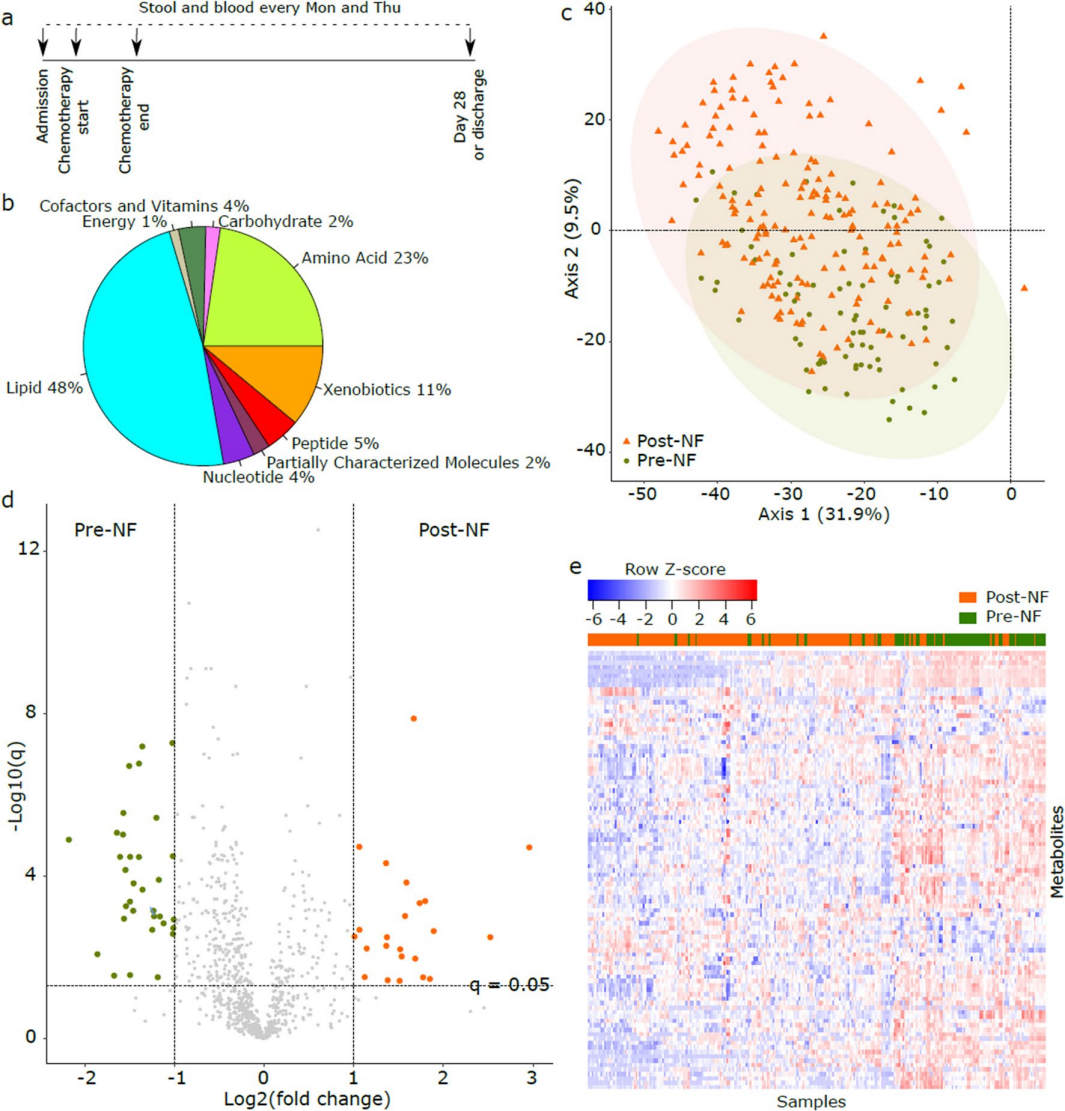
*Metabolomic changes in the serum after NF*. (**a**) Study timeline. A window of + /− 1 day was permitted for each sample. (**b**) Pathway distribution of metabolites detected in 260 serum samples from 36 patients with acute myeloid leukemia. 872 metabolites that were detectable in at least half of the samples were included. (**c**) Principal components analysis using metabolite concentrations. Pre-NF and post-NF samples in this unsupervised analysis are shown in different colors and shapes. (**d**) Volcano plot comparing metabolite concentrations between sample groups. Each point represents a metabolite. Points above the *q* = 0.05 line represent metabolites significantly associated with sample groups. *q* values were derived from per-metabolite two-sided Welch’s t-tests followed by correction for multiple testing using the Benjamini–Hochberg method. Points to the right (left) of the right (left) vertical line represent metabolites with > twofold higher concentration in post-NF (pre-NF) samples. These points were magnified for better visualization. Detailed results for significant metabolites are provided in Supplementary Data 2. (**e**) Hierarchical clustering using Euclidean distances and the complete agglomeration method for clustering. NF status was superimposed on the heatmap after the completion of clustering. Significant metabolites (*q* < 0.05) in the volcano plot (panel c) were used to generate the heatmap. Samples in panels (**c**–**e**) were classified into two groups: pre-NF (collected before NF) versus post-NF (collected after NF). NF: neutropenic fever.

In a prospective biorepository protocol (ClinicalTrials.gov: NCT03316456) approved by the University of Minnesota Institutional Review Board, we collected serum and stool samples from consecutive hospitalized adult patients with AML (newly diagnosed or relapsed/refractory) receiving chemotherapy with an expected ~ 4 weeks of hospitalization. No other inclusion or exclusion criteria were used. Sample collection started with hospital admission and continued twice weekly (Mon/Thu) until day 28 of chemotherapy or discharge (whichever occurred first) (Fig. 1a). Serum samples were collected preprandially between 6 and 8 AM in standard red-top tubes, split in 250 μL aliquots, and stored at − 80 °C within 2 h of collection. Stool samples were collected in 95% ethanol-filled sterile tubes and stored at − 80 °C. NF was defined as an oral temperature of 100.4°F combined with an absolute neutrophil count (ANC) ≤ 0.5 × 10^9^/L^20^. No specific threshold for fever duration was used to define NF. All subjects provided written informed consent and all methods were performed in accordance with the guidelines of the declaration of Helsinki.

Serum metabolome was profiled (Metabolon, Morrisville, NC) using untargeted, ultrahigh performance liquid chromatography-tandem mass spectroscopy (UPLC-MS/MS). The gut microbiota was profiled by amplicon sequencing the 16S rRNA gene (V4 hypervariable region), followed by exact amplicon sequence variant (ASV) inference using DADA2^21^ (R package *dada2* v1.18.0) and taxonomic assignment using the SILVA non-redundant v138.1 training set^22^. Raw sequence reads were uploaded to the NCBI Sequence Read Archive BioProject #SRP141394. Further methodological details for metabolome and microbiome profiling are provided in supplementary methods.

Statistical analysis was performed in R 3.4 (R Foundation for Statistical Computing, Vienna, Austria). Metabolites detectable in < 50% of all serum samples were excluded. Missing values of each remaining metabolite were imputed with the half minimum of the observed values for that metabolite, plus a small random noise generated from the Gaussian distribution to avoid ties in statistical analyses. To compare serum metabolomics before versus after NF, serum samples were classified into two groups based on whether they were collected before or after NF. Principal components analysis was used as an unsupervised algorithm to visualize metabolomic variation (package *factoextra*). Permutational analysis of variance (PERMANOVA) using Bray–Curtis distances (package *vegan*, *adonis* test with 999 permutations) was used to determine the contribution of sample groups and patient ID to metabolomic variation. Volcano plots were generated (package *ggplot2*) using the results of a two-sided Welch’s t-test comparing metabolite concentrations between the groups. Hierarchical clustering (package *gplot*, function *heatmap.2*) of serum samples was performed using Euclidean distances and the complete agglomeration method for clustering. NF status was superimposed on the heatmap after the completion of clustering. We used sparse partial least squares discriminant analysis (sPLS-DA; package *mixOmics*) to find a set of metabolites that maximize group separability of pre- versus post-NF samples^23^. Parameter tuning was done by tenfold cross validation. sPLS-DA model performance was evaluated using leave-one-out cross validation. The final set of sPLS-DA metabolites were selected using a stability threshold of 90%. These metabolites were used to define the metabolomic signature of each group. All *p* values were adjusted for multiple comparisons by the Benjamini–Hochberg method^24^ and a threshold of 0.05 for the corrected *p* values (*q* values) was used to define statistical significance.

ASVs present in < 10% of the stool samples and samples with < 5000 reads or < 1000 copies/mL of 16S rRNA gene were excluded. To estimate alpha diversity (inverse Simpson index^25^) and compare it between pre- versus post-NF samples, we used scaling with ranked subsampling (*SRS* package)^26^ with normalization to the lowest sequencing depth to adjust for sample depth variability. A Wilcoxon’s test was then used for inter-group comparison. Beta diversity was estimated using the Aitchison distance and centered log-ratio abundances^27^. Ordination was visualized using principal component analysis of the distance matrix. An adonis test with 999 permutations was used to determine the partitioning of the distance matrix between pre- and post-NF groups^28^. To investigate the microbiome-metabolome association, we paired each serum sample with its nearest preceding stool sample within 3 days. After combining all ASVs belonging to the same genus, genera present in < 20% of the samples were filtered. The final set of metabolites from sPLS-DA were normalized by rank-based inverse normal transformation (package *FRGEpistasis*, function *rankTransPheno*). A sparse linear log-contrast model^29^ was then built to identify genera (predictors) that are associated with each metabolite (response variable). This approach preserves the compositional nature of microbiome data by applying a zero-sum constraint on the compositional vector. L1 regularization, applied for variable selection, eliminates unimportant features by setting their regression coefficients to zero. We used tenfold cross-validation to optimize the tuning parameter and 100 bootstraps for stability selection analysis. The final list of important genera included those that remained in > 90 of the bootstraps.

## Results

Patient characteristics are summarized in Table 1. The most commonly used antibacterial antibiotics were levofloxacin (33 patients, 92%), 3rd or higher generation cephalosporins (30 patients, 83%), intravenous vancomycin (21 patients, 58%), piperacillin-tazobactam (17 patients, 47%), metronidazole (12 patients, 33%), and oral vancomycin (4 patients, 11%). Since 94% of patients were newly diagnosed, their previous hospitalization and antibiotic history was negligible. 13 (36%) patients required parenteral nutrition. All but 3 patients developed NF, and no patient had more than one distinct episode of NF. NF occurred at a median (range) of 6 (-3 to 20) days after starting chemotherapy. The median (range) duration of NF was 3 (range: 1–19) days. Documented infections included bloodstream infection in 17 patients (47%), *Clostridioides difficile* infection in 4 (11%) patients, pneumonia in 5 (14%) patients, and a soft tissue abscess in 1 (3%) patient.

**Table 1:** Patient characteristics

Total, N	36
Age, years	
Median (range)	60 (27–80)
Sex, n (%)	
Male	22 (61)
Female	14 (39)
Disease phase	
Newly diagnosed	34 (94)
Relapsed/Refractory	2 (6)
Chemotherapy regimen, n (%)	
7 + 3 (with or without additional agent) or Vyxeos	28 (78)
Clofarabine-based	3 (8)
Others	5 (14)
Most common antibacterial antibiotics, n (%)	
Levofloxacin	33 (92)
3rd or higher generation cephalosporins	30 (83)
Intravenous vancomycin	21 (58)
Piperacillin-tazobactam	17 (47)
Metronidazole	12 (33)
Oral vancomycin	4 (11)

7 + 3: Anthracycline + Cytarabine.

260 serum samples (pre-NF: 84, post-NF: 176) from 36 patients were available for metabolomic analysis. We detected 945 circulating metabolites, 872 of which were detectable in at least half of the samples and were stored for further analysis (Supplementary Data 1). The distribution of these metabolites into metabolic pathways is shown in Fig. 1b. Principal components analysis using metabolite concentrations suggested partial clustering of samples by NF (Fig. 1c). Over half of the variation in the metabolome was explained by whether the samples were collected before versus after NF (PERMANOVA R^2^ = 0.56, *adonis* test with 999 permutations). As patient ID did not contribute to metabolomic variance (R^2^ = 0.02), a patient-level factor was not considered in further analyses. The level of 396 metabolites was significantly different between pre- and post-NF samples (*q* < 0.05, two-sided Welch’s t-test; Fig. 1d and Supplementary Data 2). Hierarchical clustering using these 396 metabolites indicated separability of the groups (Fig. 1e).

**Figure 2:**
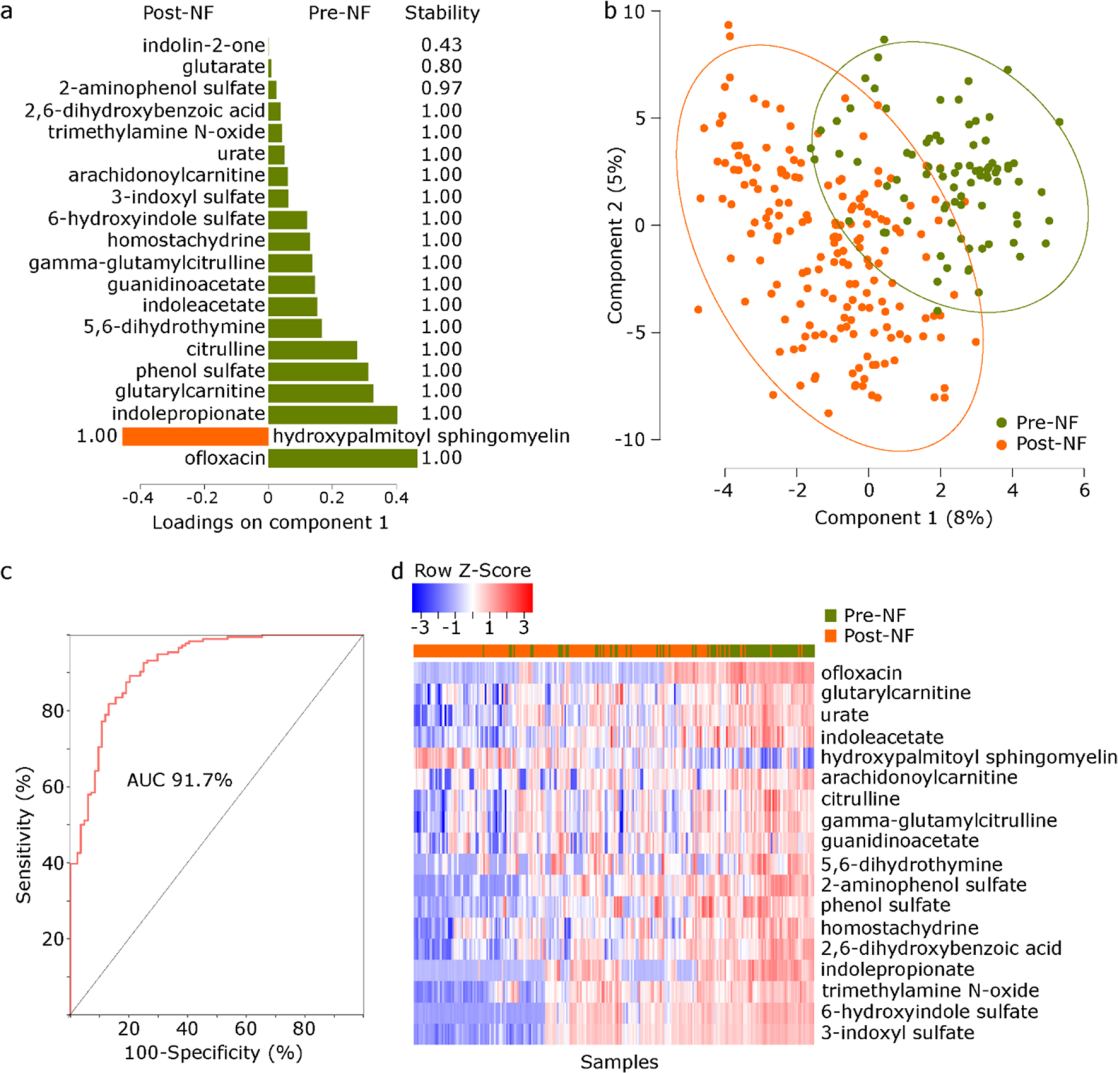
*Sparse partial least squares discriminant analysis*. (**a**) Metabolite loadings on component 1, with their stability shown next to each metabolite. Bars to the right (left) show metabolites associated with pre-NF (post-NF) samples. (**b**) Clustering of samples using metabolites on the first 2 components. (**c**) Receiver operating characteristic curve using metabolites on component 1 to predict sample groups. (**d**) Hierarchical clustering using metabolites on component 1 with > 90% stability. NF status was superimposed on the heatmap after the completion of clustering. NF: neutropenic fever.

Next, we used sPLS-DA to find the optimal linear combination of metabolites that maximized the separation of pre- versus post-NF samples. This supervised algorithm identified 20 metabolites on component 1 and 220 on component 2 as important predictors of pre- versus post-NF status. Metabolite loadings on component 1 are shown in Fig. 2a and Supplementary Data 3. All but one metabolite (a sphingomyelin metabolite) were associated with pre-NF samples. Ofloxacin, an enantiomer of levofloxacin^30^, was the metabolite most strongly associated with pre-NF samples. Because our institutional antibiotic stewardship recommends levofloxacin prophylaxis against NF and its replacement with broader-spectrum antibiotics at the time of NF, this finding supports the internal validity of the sPLS-DA approach. Pre- and post-NF samples segregated using the first 2 components of sPLS-DA (Fig. 2b). Using the 20 metabolites in component 1, the area under the curve for group classification was 91.7% (Fig. 2c). In stability analysis, the stability of 18 of these metabolites was > 90% (Fig. 2a). All but one of these metabolites (urate) were significant (*q* < 0.05) predictors of NF status in the previous analysis using Welch’s t-test. Hierarchical clustering using the 18 sPLS-DA metabolites with > 90% stability separated pre- vsersu post-NF samples, thus providing a minimal metabolomic signature for each group (Fig. 2d).

We performed two additional exploratory analyses. First, we excluded patients with bloodstream infection and compared pre- versus post-NF samples for the 18 metabolites in the previous analysis. The goal of this analysis (143 samples) was to evaluate whether these metabolites might explain fevers due to reasons other than bacteremia. The level of 17 of the 18 metabolites remained significantly different between the groups (*p* < 0.001). In the second analysis, we included only the last pre-NF and first post-NF samples for each patient and compared them for the same 18 metabolites. The goal of this analysis was to evaluate the potential effect of repeated measures on our results. Although sample size in this analysis was rather small (53 samples) and reduced our statistical power, the level of 9 of the 18 metabolites remained significantly different between the groups (*p* < 0.05). These analyses overall supported our main findings.

**Figure 3:**
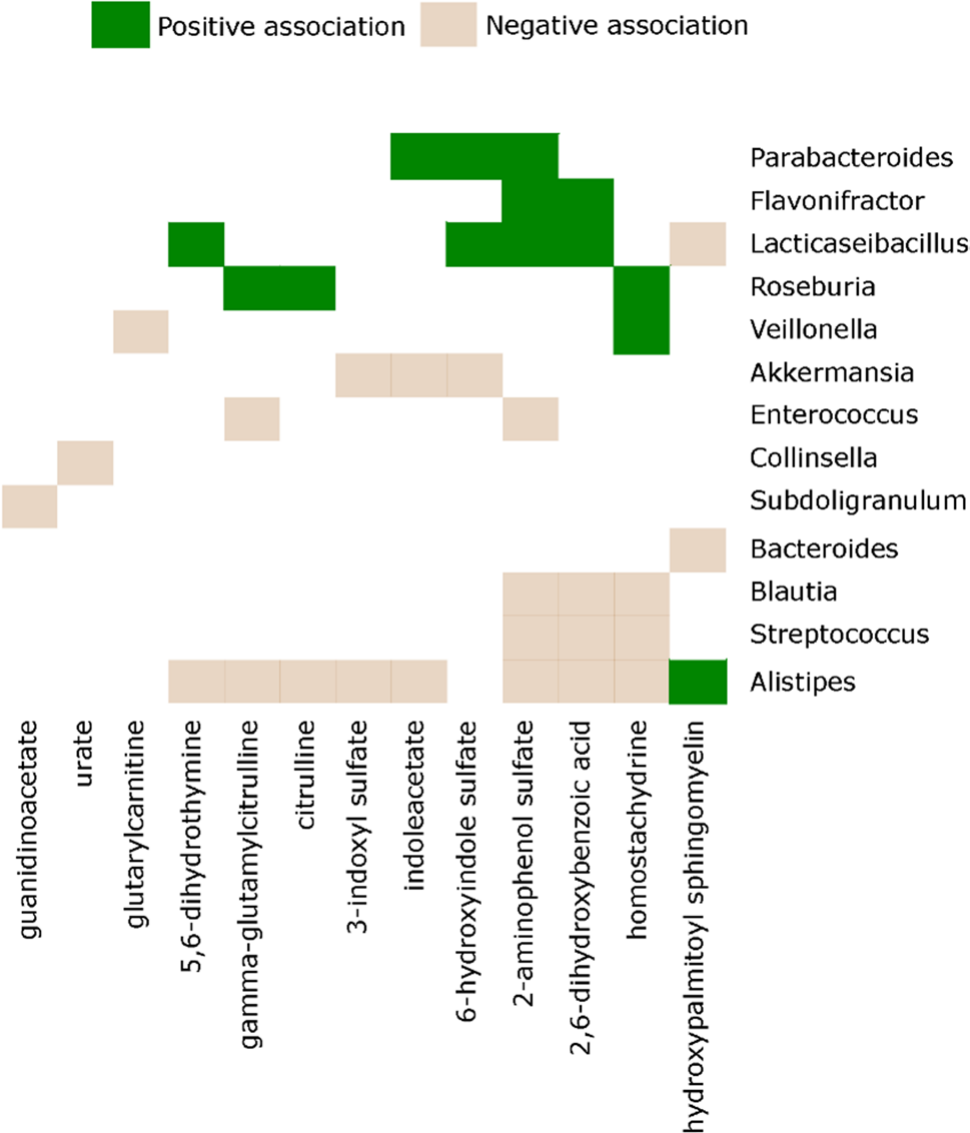
*Gut microbiota-serum microbiome association*. Sparse log-contrast modeling to find genera within the gut microbiome that predicted the final set of 18 sPLS-DA metabolites within the next 3 days. 220 paired samples were used for analysis. This analysis identified 38 stable (> 90% in 100 bootstraps) associations between 13 genera and 13 metabolites. These associations were visualized using a binarily colored plot showing the direction of the associations.

Inspired by the role of the gut microbiota in regulating circulating metabolites^9,31,32^, next we used sparse log-contrast modeling to identify genera within the gut microbiome that predicted the final set of 18 sPLS-DA metabolites within the next 3 days. We assumed that if a causal connection were present between a taxon and a metabolite, it would be present irrespective of fever status. Therefore, we included both pre- and post-NF samples. A total of 410 stool samples were collected, yielding 9,033,498 high-quality sequences and 25,110 ASVs. The median (range) number of reads per sample was 16,522 (5,193–114,449). Pre-NF samples had greater alpha diversity than post-NF samples (*p* = 0.04; supplementary Fig. 1). In beta diversity analysis, the first two principal components explained only about 20% of microbiota variation. As a result, although NF status (pre vs. post) was a significant contributor to microbiota compositional variation (adonis *p* < 0.001, 999 permutations), this was not readily apparent in the 2-dimensional ordination space (supplementary Fig. 1). After filtering rare ASVs, low-yield samples, and rare genera, we identified 20 genera in 339 samples. Of these, 220 could be paired with serum samples, yielding a total of 220 pairs for sparse log-contrast modeling. This analysis identified a final list of 38 stable associations between 13 genera and 13 metabolites (Fig. 3).

## Discussion

In this multi-omics analysis, we identified a major serum metabolomic shift after NF. A minimal metabolomic signature included 18 metabolites which distinguished pre- versus post-NF samples with a high accuracy. Of particular interest among these metabolites were 2 citrulline and 4 indole derivatives, all associated with pre-NF samples. Citrulline, an amino acid exclusively produced by intestinal epithelial cells, is a biomarker of total functioning enterocyte mass^33,34^. Indole is a metabolite of dietary tryptophan produced by specific commensal gut bacteria^35^ and absorbed into the blood. Indole metabolites augment intestinal barrier integrity and attenuate local mucosal inflammation^36,37^. The association of citrulline and indole with pre-NF samples suggests a protective role for gut barrier integrity and commensal microbiota against NF. Sphingomyelin, the only metabolite associated with post-NF samples, is a major lipid in the cell membrane. An essential component of sphingomyelin is a ceramide, known to mediate the rapid phase of febrile response to interleukin-1ß^38^. The finding that 17 of the 18 metabolites were associated with pre-NF samples and only 1 with post-NF samples suggests the pre-NF metabolome is enriched in metabolites that protect against NF. The post-NF metabolome is characterized primarily by a reduction in these protective metabolites rather than an increase in detrimental metabolites. The current approach to the prevention and treatment of NF is antimicrobial, neglecting the potential value of protective metabolites produced by the commensal microbiota.

By analyzing the serum metabolome in conjunction with the preceding gut microbiome, we found candidate gut bacteria that may mediate some of the observed metabolomic changes. The regulatory role of gut microbiota on circulating metabolites in healthy individuals has been established^31,32^. Our analytic approach explicitly considered the compositionality of microbiota data. In addition, L1 regularization combined with stability selection eliminated most spurious associations and produced a short list of stable metabolite-bacteria associations that are likely biologically important. With 38 such associations, numerous novel hypotheses may be generated for future research. As an example, both citrulline metabolites and 3 of the 4 indole metabolites from sPLS-DA were among the metabolites significantly associated with the gut microbiota. Higher abundance of *Akkermansia*, a prototype mucolytic genus, predicted lower levels of all 3 indole derivatives in the blood. We recently reported that *Akkermansia* expansion in the gut in these patients predicted a higher incidence of NF in the next several days and proposed this to be mediated by alterations in the host metabolic response^9^. Our findings in the present work support this hypothesis. Similarly, *Alistipes* is another genus that lives within or in close proximity to the mucus and uses mucin as a nutrient. This genus was inversely associated with 2 indole and 2 citrulline metabolites in the present analysis. Intriguingly, *Parabacteroides* predicted higher levels of 2 indole derivatives. If this association is validated, supplementation may be considered as a potential approach to NF prophylaxis, especially in patients who have lower levels of this genus. Another interesting association was between *Roseburia* and 2 citrulline metabolites. This genus is one of most potent butyrate producers in the human gut^39^. Butyrate and other short-chain fatty acids provide tonic stimuli for the gut epithelium^40^.

Patients with NF commonly experience non-specific symptoms including malaise, fatigue, headache, loss of appetite, irritability, confusion, sleep alterations, and muscle aches, which often result in prolonged hospitalization and poor quality of life^41^. In addition, fever can influence hematopoietic recovery after cytotoxic damage^42^. Whether the observed metabolomic changes mediate some of the fever-associated symptoms or mediate the effects of fever on hematopoietic recovery is subject to further investigation.

As diet is a regulator of the gut microbiome and serum metabolome, lack of granular dietary data is a limitation of the present study. Furthermore, the findings of this study are associational in nature. Although some of the significant metabolites have known beneficial or detrimental effects in specific settings, their causal contribution to NF in patients with AML cannot be ascertained. Therefore, mechanistic studies are necessary to prove causality. Nonetheless, our findings support a model where the gut microbiota and gut barrier cooperate to protect against NF. A breakdown of this cooperation occurs because of disruptions to the microbiota (*e.g.* due to antibiotics) and gut barrier (*e.g.* due to chemotherapy), collectively increasing host susceptibility to pyrogenic stimuli. While the current anti-microbial practice implicitly assumes that the main cause of NF is a microbe (whether or not documented) or its detrimental products, our findings highlight the importance of the protective metabolites produced by the commensal gut microbiota. Therefore, strategies that protect/restore the microbiota and augment the gut barrier could be novel approaches to the prevention and treatment of NF.

## Supplementary Information

Supplementary Information 1.

Supplementary Information 2.

Supplementary Information 3.

Supplementary Information 4.

Supplementary Information 5.

Supplementary Information 6.
