# Supplementary material for: Loss of microbiota-derived protective metabolites after neutropenic fever
Source: Sci Rep. 2022 Apr 15;12:6244. doi: 10.1038/s41598-022-10282-0 (PMC9012881; doi:10.1038/s41598-022-10282-0)
Supplement: Supplementary file 1 — Supplementary Information 1. [file 41598_2022_10282_MOESM1_ESM.pdf]

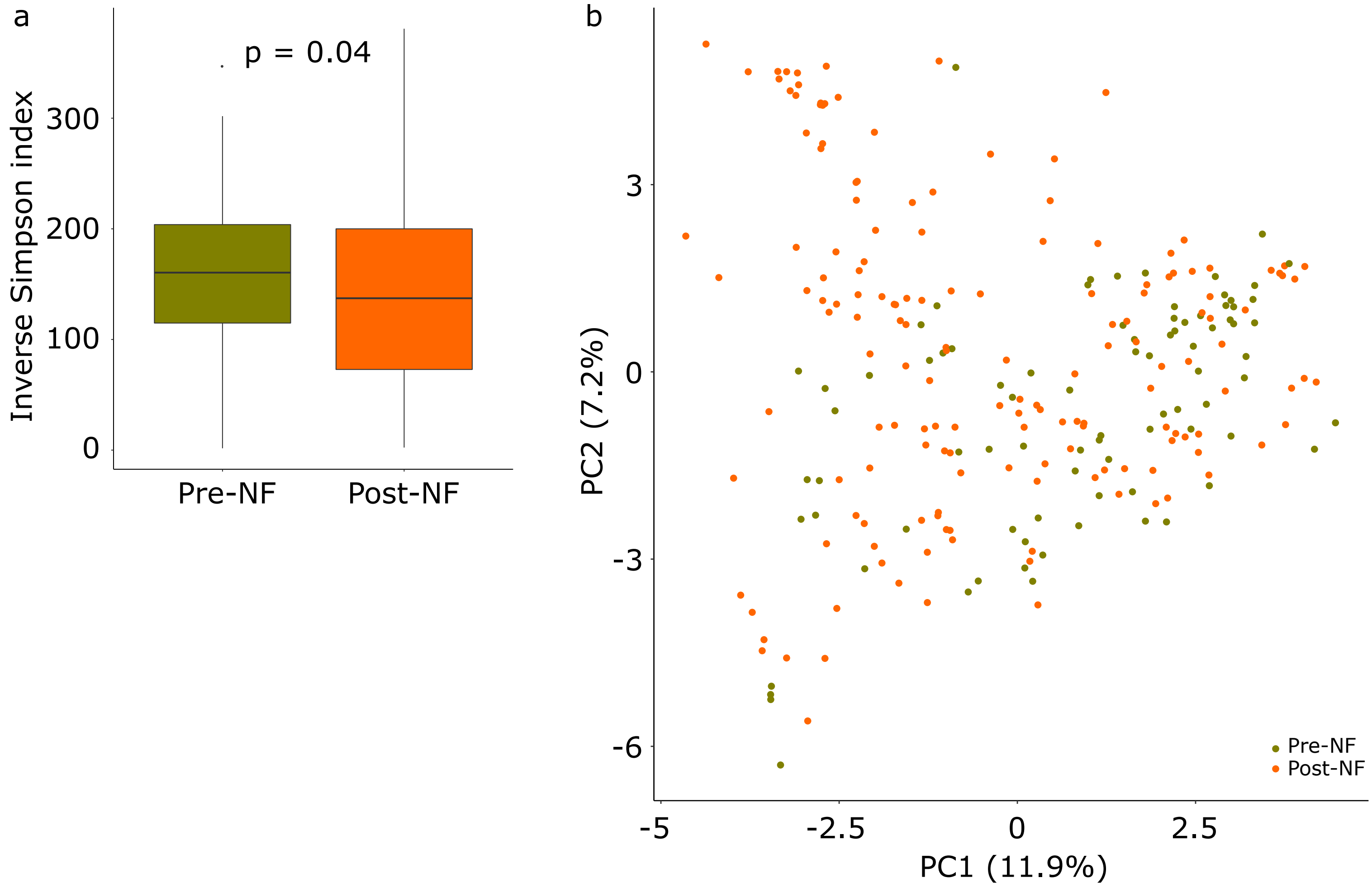

Figure S1: Alpha and beta diversity analysis

(**a**) Inverse Simpson index in samples collected before vs. after NF. The p value is from a Wilcoxon's test. (**b**) Principal component analysis using Aitchison distances and centered log-ratio transformed ASV abundances. Numbers in parentheses indicate the fraction of microbiota compositional variation explained by the corresponding axis. NF: Neutropenic fever; PC: principal component
