# Supplementary material for: Loss of microbiota-derived protective metabolites after neutropenic fever
Source: Sci Rep. 2022 Apr 15;12:6244. doi: 10.1038/s41598-022-10282-0 (PMC9012881; doi:10.1038/s41598-022-10282-0)
Supplement: Supplementary file 3 — Supplementary Information 3. [file 41598_2022_10282_MOESM3_ESM.docx]

**Supplementary methods**

**Gut microbiome profiling**

DNA was extracted from the stool samples using the DNeasy PowerSoil DNA isolation kit (QIAGEN, Hilden, Germany). The V4 hypervariable region of the 16S rRNA gene was amplified on an Illumina MiSeq platform (2 x 300 paired-end mode)[^1^](https://paperpile.com/c/g4skw6/3dHWB). Adaptor trimming was done using SHI7[^2^](https://paperpile.com/c/g4skw6/EogtN), and the resulting demultiplexed fastq files were used as input to DADA2[^3^](https://paperpile.com/c/g4skw6/ObD8C) to infer exact ASVs from amplicon data (*dada2* package v1.18.0 in R 3.4). For filtering, we used DADA2 default parameters (PHRED score threshold of 2, maximum number of expected errors of 2 for both forward and reverse reads) and truncation lengths of 220 (forward) and 150 (backward). De-replication, de-noising, merging, and chimera removal were done using DADA2 default parameters. Taxonomic assignment was done by the naive Bayesian classifier implemented in DADA2 and the SILVA non-redundant v138.1 training set[^4^](https://paperpile.com/c/g4skw6/pIuVK).

**Serum metabolome profiling**

***Sample preparation.*** Serum samples were prepared using the automated MicroLab STAR® system from Hamilton Company. For the metabolomic analysis, a total of 100 μL of sample was extracted under vigorous shaking for 2 min (Glen Mills GenoGrinder 2000) with methanol 80%, containing the following recovery standards: DL-2-fluorophenylglycine, tridecanoic acid, d6-cholesterol, and DL-4-chlorophenylalanine. The resulting extract was divided into 5 fractions: two for analysis by two separate reverse phase (RP)/UPLC-MS/MS methods with positive ion mode electrospray ionization (ESI), one for analysis by RP/UPLC-MS/MS with negative ion mode ESI, and one for analysis by HILIC/UPLC-MS/MS with negative ion mode ESI. The remaining aliquot was reserved for backup. Samples were placed briefly on a TurboVap® (Zymark) to remove the organic solvent. The sample extracts were stored overnight under nitrogen before preparation for analysis.

***Mass spectrometry.*** All methods utilized a Waters ACQUITY UPLC and a Thermo Scientific Q-Exactive high-resolution/accurate mass spectrometer interfaced with a heated electrospray ionization (HESI-II) source and Orbitrap mass analyzer operated at *R* = 35,000 mass resolution. The sample extract was dried then reconstituted in solvents compatible to each of the four methods. For each sample, two aliquots of each sample were reconstituted in 50 μL of 6.5 mM ammonium bicarbonate in water (pH 8) for the negative ion analysis and another two aliquots of each were reconstituted using 50 μL 0.1% formic acid in water (pH ~3.5) for the positive ion method. Each reconstitution solvent contained a series of standards at fixed concentrations to ensure injection and chromatographic consistency. The internal standards consist of a variety of deuterium labeled or halogenated biochemicals specifically designed both to cover the entire chromatographic run and to not interfere with the detection of any endogenous biochemicals. Authentic standards of d7-glucose, d3-leucine, d8-phenylalanine, and d5-tryptophan were purchased from Cambridge Isotope Laboratories (Andover, MA). d5-hippuric acid, d5-indole acetic acid, and d9-progesterone were procured from C/D/N Isotopes, Inc. (Pointe-Claire, Quebec). Bromophenylalanine was provided by Sigma-Aldrich Co. LLC. (St. Louis, MO) and amitriptyline was from MP Biomedicals, LLC. (Aurora, OH). Recovery standards of DL-2-fluorophenylglycine and DL-4-chlorophenylalanine were from Aldrich Chemical Co. (Milwaukee, WI). Tridecanoic acid was purchased from Sigma-Aldrich (St. Louis, MO) and d6-cholesterol was from Cambridge Isotope Laboratories (Andover, MA). Standards for the HILIC dilution series of alpha-ketoglutarate, ATP, malic acid, NADH, and oxaloacetic acid were purchased from Sigma-Aldrich Co. LLC. (St. Louis, MO) while succinic acid, pyruvic acid and NAD+ were purchased from MP Biomedicals, LLC. (Santa Ana, CA). Limit of detection (LOD) for standards analyzed in a dilution series using reverse phase chromatography is available in **Table 1**.

**Table 1:** Limit of detection (LOD) for standards in a dilution series using reverse-phase chromatography

| **Standard** | **HRAM LOD ng/mL** | **UMR LOD ng/mL** |
| --- | --- | --- |
| d7-glucose | 1.0 | 50.0 |
| d3-leucine | 0.25 | 5.0 |
| d8-phenylalanine | 0.25 | 3.0 |
| d5-tryptophan | 0.25 | 25.0 |
| d5-hippuric acid | 0.25 | 5.0 |
| Br-phenylalanine | 0.25 | 3.0 |
| d5-indole acetic acid | 3.0 | 25.0 |
| amitriptyline | 0.5 | 3.0 |
| d9-progesterone | 1.0 | 25.0 |

One aliquot was analyzed using acidic positive ion conditions (LC pos), chromatographically optimized for more hydrophilic compounds. In this method, the extract was gradient eluted from a C18 column (Waters UPLC BEH C18-2.1 × 100 mm, 1.7 µm) using water and methanol, containing 0.05% perfluoropentanoic acid (PFPA) and 0.1% formic acid (FA) at pH = 2.5. Elution was performed at 0.35 mL min−1 in a linear gradient from 5% to 80% of methanol containing 0.1% FA and 0.05% PFPA over 3.35 min. A second aliquot was also analyzed using acidic positive ion conditions; however, it was chromatographically optimized for more hydrophobic compounds. In this method, the extract was gradient eluted from the same aforementioned C18 column using methanol 50%, acetonitrile 50%, water, 0.05 % PFPA, and 0.01 % FA at pH = 2.5 and was operated at an overall higher organic content. Elution was performed at 0.60 mL/min in a linear gradient from 40% to 99.5% over 1 min, hold 2.4 min at 99.5% of methanol 50%, acetonitrile 50%, 0.05% PFPA, and 0.01% FA. A third aliquot was analyzed using basic negative ion-optimized conditions with a separate dedicated C18 column (LC neg). The basic extracts were gradient eluted from the column using methanol 95% and water 5%, with 6.5 mM ammonium bicarbonate at pH 8. Elution was performed at 0.35 mL min−1 with a linear gradient from 0.5% to 70% of methanol 95%, water 5% with 6.5 mM ammonium bicarbonate over 4 min, followed by a rapid gradient to 99% in 0.5 min. The sample injection volume was 5 μL and a 2× needle loop overfill was used. Separations utilized separate acid and base-dedicated 2.1 mm × 100 mm Waters BEH C18 1.7 μm columns held at 40 °C. The fourth aliquot was analyzed via negative ionization following elution from an HILIC column (LC HILIC) (Waters UPLC BEH Amide 2.1 × 150 mm, 1.7 µm, held at 40 °C) using a gradient consisting of water (15%), methanol (5%), and acetonitrile (80%) with 10 mM ammonium formate, pH 10.16. Elution flow rate was 0.5 mL/min with a linear gradient from 5% to 50% in 3.5 min, followed by a linear gradient from 50% to 95% in 2 min, of water (50%), acetonitrile (50%) with 10 mM ammonium formate, pH 10.6. The MS analysis alternated between MS and data-dependent MSn scans using dynamic exclusion. The scan range varied slightly between methods but covered 70–1000 *m/z*.

***Quality assurance and quality control (QA/QC).*** Several types of controls were analyzed in concert with the experimental samples: a pooled matrix sample generated by taking a small volume of each experimental sample served as a technical replicate throughout the platform run; extracted water samples served as process blanks; and a cocktail of QC standards (carefully chosen not to interfere with the measurement of endogenous compounds) spiked into every analyzed sample allowed instrument performance monitoring and aided chromatographic alignment. **Tables 2-4** describe QC samples and standards. Instrument variability was determined by calculating the median relative standard deviation (RSD) for the internal standards that were added to each sample prior to injection into the mass spectrometers (median RSD = 3–4%). Instruments are calibrated at least weekly in the utilized polarity using thermo and mass accuracy is monitored at the batch level for the internal standards. A batch fails QC if any of the internal standards are more than 5 ppm away from the theoretical mass. Overall process variability was determined by calculating the median RSD for all endogenous metabolites (i.e., non-instrument standards) present in 100% of the pooled matrix (MTRX) samples, which are technical replicates created from a large pool of extensively characterized human plasma. The median RSD for MTRX samples was 9–10%. Five MTRX samples and three process blank samples were processed per every batch of 30 samples. Experimental samples were randomized across the platform run with QC samples spaced evenly among the injections, as outlined in **Figure 1** below.

**Table 2:** Description of metabolon QC samples

| **Type** | **Description** | **Purpose** |
| --- | --- | --- |
| CMTRX | Pool created by taking a small aliquot from every customer samples. | Assess the effect of a non-plasma matrix on the Metabolon process and distinguish biological variability from process variability. |
| PRCS | Aliquot of ultra-pure water | Process Blank used to assess the contribution to compound signals from the process. |
| SOLV | Aliquot of solvents used in extraction. | Solvent Blank used to segregate contamination sources in the extraction. |

**Table 3:** Metabolon QC standards

| **Type** | **Description** | **Purpose** |
| --- | --- | --- |
| RS | Recovery Standard | Assess variability and verify performance of extraction and instrumentation. |
| IS | Internal Standard | Assess variability and performance of instrument. |

**Table 4:** Quality control internal standards

| **Condition** | **Internal standards** |
| --- | --- |
| LC neg | d7-glucose  d3-methionine  d3-leucine  d8-phenylalanine  d5-tryptophan  Br-phenylalanine  d15-octanoic acid  d19-decanoic acid  d27-tetradecanoic acid  d35-octadecanoic acid  d2-eicosanoic acid |
| LC HILIC | d35-octadecanoic acid  d5-indole acetic acid  Br-phenylalanine  d5-tryptophan  d4-tyrosine  d3-serine  d3-aspartic acid  d7-ornithine  d4-lysine |
| LC pos | d7-glucose  d3-methionine  d3-leucine  d8-phenylalanine  d5-tryptophan  Br-phenylalanine  d4-tyrosine  d5-indole acetic acid  d5-hippuric acid  amitriptyline  d9-progesterone  d4-dioctylphthalate |


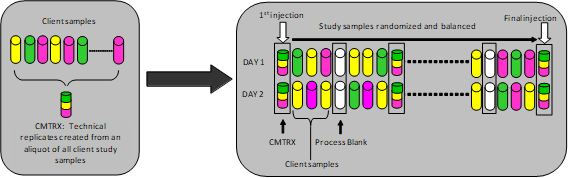


**Figure 1. Preparation of client-specific technical replicates**. A small aliquot of each client sample (colored cylinders) is pooled to create a CMTRX technical replicate sample (multi-colored cylinder), which is then injected periodically throughout the platform run. Variability among consistently detected biochemicals can be used to calculate an estimate of overall process and platform variability.

All studies include the analysis of a technical replicate of a sample pooled from the experimental samples. This pool was analyzed 16 times over the course of the analysis of the experimental samples in the present study.

***Compound identification and quantification.*** Raw data were extracted, peak-identified, and QC processed using Metabolon pipelines. Compounds were identified by comparison to library entries of purified standards or recurrent unknown entities[^5,6^](https://paperpile.com/c/g4skw6/bq2nd+4vAyW). Briefly, Metabolon maintains a library based on authenticated standards (analyzed using the same methodology as the experimental samples) that contains the retention time/index (RI), mass to charge ratio (*m/z)*, and chromatographic data (including MS/MS spectral data) on all molecules present in the library. Furthermore, biochemical identifications are based on three criteria: retention index within a narrow RI window of the proposed identification (typically within a 5 second window), accurate mass match to the library ±10 ppm (typically well within a 5 ppm window), and the MS/MS forward and reverse scores between the experimental data and authentic standards. The MS/MS scores are based on a comparison of the ions present in the experimental spectrum to the ions present in the library spectrum. While there may be similarities between these molecules based on one of these factors, the use of all three data points can be utilized to distinguish and differentiate biochemicals. More than 3300 commercially available purified standard compounds have been acquired and registered for analysis on all platforms for determination of their analytical characteristics. Compounds in **supplementary data 1** for which no authentic standards were available for confirmation are marked with an “*” after the compound name to designate these as Metabolomics Standards Initiative level 2/3[^7^](https://paperpile.com/c/g4skw6/PgoDL). Most such identifications are based on the experimental signature having the same characteristics as the compound class. For example, sphingomyelins all have a conserved fragmentation spectrum and so have a highly diagnostic pattern in order to permit the identification of the experimental signature as a sphingomyelin. The QC and curation processes were designed to ensure accurate and consistent identification of true chemical entities, and to remove those representing system artifacts, mis-assignments, and background noise. Metabolon data analysts use proprietary visualization and interpretation software to confirm the consistency of peak identification among the various samples. Library matches for each compound were checked for each sample and corrected if necessary. Peaks were quantified using area-under-the-curve. A data normalization step was performed to correct variation resulting from instrument inter-day tuning differences. Essentially, each compound was corrected in run-day blocks by registering the medians to equal 1 and normalizing each data point proportionately. Metabolites were assigned to pathways based on three publicly available key chemical information resources: PubChem, HMDB and KEGG pathway database.

**References**

1. [Gohl, D. M. *et al.* Systematic improvement of amplicon marker gene methods for increased accuracy in microbiome studies. *Nat. Biotechnol.* **34**, 942–949 (2016).](http://paperpile.com/b/g4skw6/3dHWB)

2. [Al-Ghalith, G. A., Hillmann, B., Ang, K., Shields-Cutler, R. & Knights, D. SHI7 Is a Self-Learning Pipeline for Multipurpose Short-Read DNA Quality Control. *mSystems* **3**, (2018).](http://paperpile.com/b/g4skw6/EogtN)

3. [Callahan, B. J. *et al.* DADA2: High-resolution sample inference from Illumina amplicon data. *Nature Methods* vol. 13 581–583 (2016).](http://paperpile.com/b/g4skw6/ObD8C)

4. [Quast, C. *et al.* The SILVA ribosomal RNA gene database project: improved data processing and web-based tools. *Nucleic Acids Res.* **41**, D590–6 (2013).](http://paperpile.com/b/g4skw6/pIuVK)

5. [DeHaven, C. D., Evans, A. M., Dai, H. & Lawton, K. A. Organization of GC/MS and LC/MS metabolomics data into chemical libraries. *J. Cheminform.* **2**, 9 (2010).](http://paperpile.com/b/g4skw6/bq2nd)

6. [DeHaven, C. D., Evans, A. M., Dai, H. & Lawton, K. A. Software techniques for enabling high-throughput analysis of metabolomic datasets. *Metabolomics* **10**, 167–192 (2012).](http://paperpile.com/b/g4skw6/4vAyW)

7. [Sumner, L. W. *et al.* Proposed minimum reporting standards for chemical analysis Chemical Analysis Working Group (CAWG) Metabolomics Standards Initiative (MSI). *Metabolomics* **3**, 211–221 (2007).](http://paperpile.com/b/g4skw6/PgoDL)
